# The Use of Macro, Micro, and Trace Elemental Profiles to Differentiate Commercial Single Vineyard Pinot noir Wines at a Sub-Regional Level

**DOI:** 10.3390/molecules25112552

**Published:** 2020-05-30

**Authors:** Courtney K. Tanabe, Jenny Nelson, Roger B. Boulton, Susan E. Ebeler, Helene Hopfer

**Affiliations:** 1 Department of Viticulture & Enology, University of California, Davis, CA 95616, USA; cktanabe@ucdavis.edu (C.K.T.); jenny.nelson@agilent.com (J.N.); rbboulton@ucdavis.edu (R.B.B.); seebeler@ucdavis.edu (S.E.E.); 2 Food Safety & Measurement Facility, University of California, Davis, CA 95616, USA; 3 Department of Food Science, The Pennsylvania State University, University Park, PA 16802, USA

**Keywords:** pinot noir wine, elemental profiling, sub-regional differences, authenticity, food safety

## Abstract

The compositional authentication of wine is of great interest, as the geographic origin of the grapes is often associated with quality, uniqueness, and authenticity. Previous elemental fingerprinting studies mainly discriminated wines from different countries or regions within a country. Here, we report the use of element profiles to distinguish commercial Pinot noir wines from five sub-regional appellations or neighborhoods within one American viticultural area (AVA). Fifty-three single cultivar wines were collected over two harvests and analyzed using microwave plasma-atomic emission spectroscopy (MP-AES) and inductively coupled plasma-mass spectrometry (ICP-MS). Of 62 monitored elements that were quantified with fully validated methods, 24 and 32 elements differed significantly across the neighborhoods and vintages, respectively (*p* < 0.05). Targeted canonical variate analysis (CVA) explained 85–90% of the variance ratio across the two vintages, indicating persistent and stable elemental fingerprints of wines at a sub-regional level. A sixth, newly founded neighborhood was correctly grouped separately from the others using a Soft Independent Modeling of Class Analogy (SIMCA), indicating the potential of elemental fingerprints for wine authenticity.

## 1. Introduction

History has long-attributed wine sensory properties to the vineyard sites on which the grapes were grown. The concept of appellations and defined districts based on geography is also central to authenticity and truthful labeling and can be traced back to the establishment of the demarcated Douro Region of Portugal in 1756 [1]. Such regulations are now widely used throughout Europe and have similarly been developed by almost all wine-producing countries in the world, including the United States, which established its first American viticultural area (AVA) in 1980 [2].

Today, there are 246 AVAs in the United States, of which 139 are located in California, with some of these AVAs overlapping or existing as smaller AVAs within larger AVAs [3]. The Oakville AVA, within the Napa Valley AVA, or the Santa Ynez Valley AVA, within the Central Coast AVA, are examples of this. 

The criteria for establishing an AVA are based on “distinguishing features”, which often include geographic qualities such as rainfall basins, river valleys, mountain ranges, soil types, climate, or administrative boundaries [2]. The location of a vineyard is also an important factor in purchase decisions [4], with consumers often willing to pay a premium for wines made from grapes from certain regions [5]. Researchers, wine collectors and consumers, and international trade agencies are becoming increasingly interested in the ability to authenticate the geographic origin of wine or food products in general. The notion of a chemical fingerprint is well-established, and chemical measurements are most commonly used in combination with multivariate statistical data analysis methods to segregate or cluster food items [6,7]. Similarly, for wine, attempts to develop robust authentication methods have been made using a variety of chemical analytes to distinguish the origin. Research efforts have shifted towards the applications of elements and elemental fingerprints, as well as isotope ratios, in determining wine authenticity, in part due to their chemical stability and independence of oxidation and aging reactions. Isotopic analyses such as D/H, ^13^C/^12^C, and ^18^O/^16^O but, also, ^206^Pb/^207^Pb, ^208^Pb/^206^Pb, and ^87^Sr/^86^Sr have shown great promise in authenticity studies; however, the limited precision of the standard quadrupole-mass spectrometer necessitates more expensive high-resolution mass spectrometry for such analyses [8,9]. Besides the need for isotopic databases, such as the EU Wine Isotopic databank, that measurements can be compared to, it is unclear how potential isotopic fingerprints will change due to the changing climate [10,11].

A wide range of element concentrations, including macro, micro, and trace levels, are found in wine, including Ca, K, Na, and Mg at concentrations between 10–1000 mg/kg and Fe, Cu, Mn, Rb, Al, and others at levels between 0.1–10 mg/kg, as well as trace elements such as Ba, Cd, Li, Ni, Pb, and V (0.1–1000 µg/kg), and have all been reported in wine authenticity studies [12]. Analyzing all of these elements requires a combination of analytical element techniques for quantification across the eight orders of magnitude in concentrations that are observed. Studies using only inductively coupled plasma-optical emission spectroscopy (ICP-OES) [13], or other spectroscopy techniques, have been limited to major, and some micro-level, elements. By combining spectroscopic instruments, such as ICP-OES, with the low-detection limits of ICP-mass spectrometry (ICP-MS), a more complete set of elements can be utilized in wine discrimination [14,15,16,17,18,19,20]. Investigations relying specifically on ICP-MS either utilized different sample dilutions in order to analyze a more complete element profile [21,22] or limited the studies to trace any minor level elements [23,24,25].

Element profiles found in wine are believed to be related to the soil and environmental conditions where the grapes are grown; however, numerous studies have demonstrated that certain factors can alter the elemental content of a wine, such as processing [26,27,28], grape cultivar [22], wine style [15,18], vintage [15,29], and contamination [30]. Nevertheless, multiple studies have successfully distinguished the geographic origin of wine, such as elemental differences in wines made from of grapes grown in different countries [13,21,24,31] or the distinctions in wines originating from different regions within countries such as Canada [27,32], Slovenia [19], Italy [20,33], Spain [24], Germany [14,34], Portugal [17,35], New Zealand [36], Australia [15], the Czech Republic [16,30], South Africa [23,37,38], Chile [18,39], Romania [25,29], China [40], Brazil [41], and the United States [42]. The success of the authentication based on elemental fingerprints in these studies varied, possibly due to the analysis of different element profiles, wine styles, and winemaking practices and, possibly, the cultivars studied.

A few studies took a step further and attempted to discriminate intraregional wines from Spain (Rioja Region) [22], New Zealand (Hawkes’ Bay Region) [36], South Africa (Stellenbosch Region) [23], Slovenia (Primorska and Posavje Regions) [19], and Australia (zones in Western Australia and Victoria) [15]. The close geographical proximity of these wines, originating from the same region, was believed to make it difficult to differentiate wines using multielement composition. The likelihood of similar climates, soil types, and overall geology meant that differences in the elements traditionally used in distinguishing wines would not exist. However, the referenced studies found that it was possible to discriminate intraregional wines using elemental fingerprints, with Ba, Cs, Cu, Mg, Mn, Ni, Rb, Sr, and Zn reported in at least three of the listed studies as being discriminating between regions [15,19,22,23,36]. 

To our knowledge, there has never been an attempt to discriminate wines produced in a single AVA in California or the United States. This study applied an elemental analysis to investigate the geographic authenticity of single cultivar (Pinot noir) of wines, originating from five neighborhoods within the Russian River Valley AVA in Northern California, over two vintages (2015 and 2017). The Russian River Valley AVA was chosen, because it contains a number of sub-regions known as neighborhoods, including the Green Valley, Laguna Ridge, Middle Reach, Santa Rosa Plain, and Sebastopol Hills (https://russianrivervalley.org/discover/neighborhoods). Further, this study complements a winemaker-driven initiative to uncover regional differences of Pinot noir wines as impacted by the vineyard location within the Russian River Valley AVA [43]. Pinot noir wines from the Russian River Valley AVA are renown worldwide, and Pinot noir is the most widely planted red grape cultivar in the AVA. 

We hypothesize that the diversity in the climate, geology, and topology in this large AVA (~ 169,000 acres) will result in detectable and stable elemental differences in the grapes and the subsequent wines produced in the different neighborhoods, which in turn can be distinguished using multivariate statistical methods (multivariate analysis of variance (MANOVA) and canonical variate analysis (CVA)). Furthermore, we also tested if the elemental fingerprint of a new neighborhood, Eastern Hills, differed significantly from the existing neighborhoods as determined by the Soft Independent Modeling of Class Analogies (SIMCA) to further validate these elemental fingerprints. 

## 2. Results and Discussion

### 2.1. Method Validation and Overall Results

Overall, 60 and 62 elements were quantified in the samples from the 2015 and 2017 harvests by MP-AES and ICP-MS. Despite the small differences in analytical methodology, the use of certified reference materials ensured comparability across the two years.

Of the 60 elements that were quantified by MP-AES and ICP-MS in the wines from the 2015 harvest, a total of 24 elements showed (i) satisfying recoveries (± 20%), (ii) were detected in at least one neighborhood above the lowest calibration point, and also, (iii) differed significantly among the five neighborhoods (*p* < 0.05; Table 1). ICP-MS and MP-AES calibration curves all showed high linearity, with R^2^ between 0.9994–1.0000 over the calibrated range. Overall, these results indicate that the used methods produced valid, accurate, and precise results (Supplementary Table S1).

For the 2017 harvest, 32 of the 62 monitored elements met the same criteria (i–iii listed above) and are reported in Table 2. The calibrated range obtained on the ICP-MS for significant elements ranged between 0.9997–1.0000 R^2^ (Supplementary Table S1).

Overall, all macrolevel elements in the mg/kg range, such as K, P, Mg, S, Ca, Si, B, Mn, and Rb, varied significantly among neighborhoods in both vintages, except for P (significant differences only in 2015) and Mg and Fe (significant differences only in the 2017 vintage). Sulfur was only measured in the 2017 vintage. For most of the microlevel elements, i.e., those with concentrations in the μg/kg range, (including Sr, Ba, Fe, Al, Ni, Cu, Ti, Pb, Li, Cr, Mo, and Cs), similar differences between neighborhoods could be detected in both vintages. Exceptions were Cr, Cu, Ti, Fe, and Al, which differed significantly in concentrations between the neighborhoods only in the 2017 vintage, and Cs and Ba, for which a significant neighborhood effect was only detected for the 2015 vintage. Significant neighborhood differences in Co in 2015 and As and Zr in 2017 could not be compared across the vintages, as these elements were only measured accurately in one of the two vintages.

Last, for the trace elements, with concentration levels below 1 μg/kg, five elements (V, Sb, Ce, Nd, and W) showed consistent neighborhood differences across both vintages, while the other nine elements could be quantified in only one of the two analysis years.

Taken together, the elemental differences in the wines originating from the different neighborhoods are generally stable across vintages, indicating consistent overall elemental fingerprints.

### 2.2. Wine Elemental Fingerprints Differ Significantly by Neighborhood and Over Two Separate Vintages

For both the 2015 and 2017 vintage, the elements listed in Table 1 and Table 2 differed significantly (*p* < 0.05) among the neighborhoods as assessed by multi- and univariate analyses of variance (MANOVA and ANOVA). There were elements that were significantly different for both harvest years, such as Li, B, V, Co, Ni, Rb, Sr, Mo, Ce, Nd, Pb, K, Ca, and Si. These elements have been reported previously in interregional elemental fingerprinting studies [15,19,22,23,36]. However, in all cases with the exception of Ca, the rank order of elemental concentrations was not the same across neighborhoods and vintages, indicating multiple underlying mechanisms that could lead to the observed differences. One possible reason for this occurrence might be that different samples were collected from wineries that utilized grapes from different vineyards in the neighborhood to produce the wines. These differences in elemental contents could also suggest a vintage effect; however, with the other variables such as rootstock and vine age not being held constant, this cannot be determined for the current study. 

**Figure 1:**
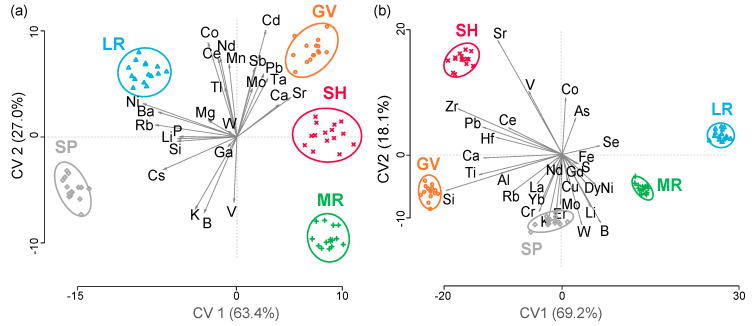
Canonical variate analysis biplots for the (**a**) 2015 vintage and (**b**) 2017 vintage, showing wine samples classified by neighborhoods (Green Valley: GV, Laguna Ridge: LR, Middle Reach: MR, Santa Rosa Plains: SP, and Sebastopol Hills: SH), with the 95% confidence circles around each neighborhood. Discriminating elements are shown as arrows.

All elements showing a significant neighborhood effect were subsequently used in the canonical variate analysis (CVA), which was carried out separately for the 2015 and 2017 harvest samples (Figure 1a,b).

For the 2015 vintage (Figure 1a), 90% of the variance ratio was explained within the first two dimensions, where wine samples were separated by sub-appellation of origin or neighborhood. Along canonical variate 1 (CV 1), explaining 63% of the variance ratio, the neighborhoods Green Valley (GV), Sebastopol Hills (SH), and Middle Reach (MR) were separated from the neighborhoods of Laguna Ridge (LR) and Santa Rosa Plains (SP). The elements driving this separation were Ni, Ba, Rb, P, Li, and Si, which were loaded negatively on CV 1 and present at significantly higher concentrations in the LR and SP neighborhoods, while Ca and Sr were loaded positively on CV 1. Calcium levels were significantly higher in the Green Valley (GV) wines compared to all other sub-regions, while wines from Sebastopol Hills (SH) showed the highest Sr levels, significantly above the levels found in all the other wines (Table 1).

Santa Rosa Plains (SP) and Middle Reach (MR) neighborhoods separated from Sebastopol Hills (SH), Laguna Ridge (LR), and Green Valley (GV) areas along the second dimension (CV 2), mostly driven by differences in V, B, and K, which were significantly higher in the Santa Rosa Plains (SP) and Middle Ridge (MR) wines, and levels of Co, Ce, Nd, Mn, Cd, Sb, Mo, Ta, and Pb, which were loaded positively on CV 2 and higher in the Laguna Ridge (LR), Green Valley (GV), and Sebastopol Hills (SH) neighborhoods.

A similar separation of neighborhoods based on wine elemental fingerprints was also achieved for the 2017 vintage, where a total of 87% of the variance ratio was captured in the first two dimensions (Figure 1b). Along CV 1, a clear separation of the wines from the Laguna Ridge (LR) and Middle Reach (MR) neighborhoods from all others was driven mostly by differences in Si, for which concentrations found in the wines were significantly lower in the wines from these two neighborhoods (18.6 and 21.7 mg/kg, respectively) compared to the neighborhoods Green Valley (GV); Santa Rosa Plains (SP); Sebastopol Hills (SH) (28.5, 26.3, and 25.8 mg/kg); and Eastern Hills (EH) (34.1 mg/kg) wines (Table 2). Further, the elemental differences in Ti, Hf, Ca, Zr, Pb, Sr, Rb, and Ce were driving the separation between the Green Valley (GV) and Sebastopol Hills (SH) neighborhoods from Laguna Ridge (LR) and Middle Reach (MR), while the latter two neighborhoods were characterized by higher concentrations of B, Li, Se, Ni, and Cu. Separation of Sebastopol Hills (SH) and, to some degree, Laguna Ridge (LR) from the other neighborhoods along the second dimension, capturing 18% of the total variance ratio, was driven mostly by differences in Sr, V, Co, As, and Zr on the positive axis and Li, B, Cr, Rb, Mo, Er, Yb, W, and K on the negative axis. 

Overall, distinct elemental fingerprints were found across both vintages, separating all neighborhoods from each other. These results provide further evidence for distinct geographical wine regions within the Russian River Valley AVA, in addition to sensory differences experienced and explored by the Neighborhood Initiative, a group of Russian River Valley winemakers looking into regional branding of the different neighborhoods. 

The analyzed wine samples were collected from commercial wineries, and although winemaking procedures were similar, they were not identical and, thus, encompassed a larger heterogeneity in the elemental composition of wines than previously explored in wine authenticity studies [31]. At the same time, the collection of wine samples onsite, directly from fermentation vessels, provided much more experimental control and authenticity than studies that analyzed commercially produced wines purchased in supermarkets, where traceability was less certain [13,17,18,21,32]. 

Several rare earth elements (REEs) differed significantly between the neighborhoods in both vintages, including La, Ce, Nd, Gd, Dy, Er, and Yb. Although the literature reports the use of rare earth elements for the geographical determination of origin (e.g., [44,45]), it was also shown that the REE content can be easily modified by various winemaking procedures, such as fining and filtering (e.g., [26,28,34,46]). More research is needed to determine the stability of REE fingerprints for processed beverages such as wines.

As seen in Table 1 and Table 2, different elements were responsible for the separation of neighborhoods across the two seasons. Nevertheless, other studies identified a similar suite of elements to distinguish sub-regional wines. For example, Angus et al. distinguished wines from one winegrowing district from wines produced in the rest of the growing region in New Zealand, using the elements Ba, Cs, Rb, and Pb [36], with cross-validation results of 92–94% accuracy in wine classification. Intraregional wine samples from different Rioja zones in Spain were distinguished by Perez-Alvarez et al. using Sr, Ba, Ni, and Cu [22], and the discriminant functions were able to classify 100% of the samples into the correct zones. Similarly, wines from four regions within Western Australian were also discriminated using Si, Rb, Er, Na, Sb, Ba, Be, Bi, and Te [15]. Validation of these sub-regional samples were correctly classified only 75% of the time, however while validating wines from three regions in Victoria, the classification increased to 87%, as Victorian wines were mainly distinguished by Li, Rb, Se, Cs, and Si contents [15].

Last, Coetzee et al. similarly found that variations in the concentration levels of B, Ba, Cs, Cu, Mg, Rb, Sr, Tl, and Zn were good indicators to distinguish different cultivars of red and white wines from different estates within a single wine district in South Africa [23]. The study showed a range of cross-validation results (30–80%) depending on the location of the estates. The results were found to be correlated to elements in the soil originating in different soil types, and a similar mechanism would be plausible to explain the differences found in our results. Besides differences in climatic factors, such as the temperature, the neighborhoods are described to differ in soil type, from shale, sandstone, and clay soils in the Santa Rosa Plains to Goldridge and Altamont soils in the Laguna Ridge, Green Valley, and Sebastopol Hills and volcanic and sedimentary soils in the new neighborhood of Eastern Hills [43]. 

The uptake of minerals by grapevine roots is known to involve several transport systems. One of these, an ATPase proton-monovalent cation exchanger, is responsible for the uptake of group 1 elements (Li, Na, K, Rb, Cs, and Fr). While potassium is the major element in this group taken up in many soils, different rootstocks and a lower availability of potassium would be expected to give rise to different concentrations in the others. This was born out in several previous studies [15,22,23,36] and is also found here. Future studies might include the grouping of elements by their rootstock transport system to look for the plant-soil interactions that appear to be driving the elemental composition of grapes and their wines.

### 2.3. A New Neighborhood Was Classified as Falling Outside the Existing Neighborhoods Based on Its Elemental Fingerprints

Among the 2017 samples, wines from a new neighborhood, Eastern Hills (EH), were included in the analysis. Elemental differences in Al, Mn, Ni, Zr, Mg, Si, Yb, Er, Cr, and S were found to be discriminating EH wines from the other neighborhoods; except for S, all listed elements were the highest in concentration in the EH samples. 

Using this new neighborhood, we were able to further validate the distinctiveness and uniqueness of the neighborhood elemental fingerprints. We first included the EH data into the 2017 data for visual statistical interpretation using CVA and then Soft Independent Modeling of Independent Classes (SIMCA) as a multiclass classification method that is able to classify unknown samples as being either within a class or outside. Samples from the new neighborhood EH should be classified by the SIMCA algorithm as originating from none of the five original neighborhoods, if this new neighborhood is indeed characterized by a unique elemental fingerprint. 

First, half of the 2017 dataset with the original five neighborhoods (Green Valley: GV, Laguna Ridge: LR, Middle Reach: MR, Sebastopol Hills: SH, and Santa Rosa Plain: SP) was used to establish and calibrate the model (α = 0.05; γ = 0.01). Then, the second half of the dataset, with the addition of the Eastern Hills (EH) samples, was then used to test the developed model (Table 3).

**Figure 2:**
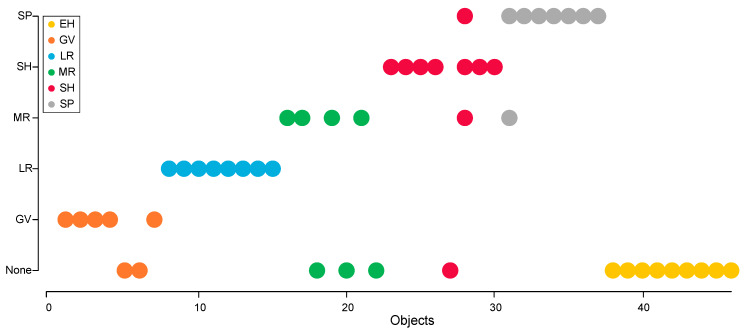
Soft Independent Modeling of Class Analogy (SIMCA) prediction pot for the six neighborhoods (Green Valley: GV, Laguna Ridge: LR, Middle Reach: MR, Santa Rosa Plains: SP, and Sebastopol Hills: SH), including the new neighborhood, Eastern Hills (EH). Each wine sample is color-coded by the originating neighborhood.

A visual presentation of the prediction models for each wine sample is shown in Figure 2 using the number of optimal components for each neighborhood class model. In some cases, the model worked well, e.g., for wines from the Laguna Ridge (LR) and Santa Rosa Plains (SP) neighborhoods, where all or all but one wine samples were correctly classified into the corresponding group. The model was less accurate for the Sebastopol Hills (SH), Middle Reach (MR), and Green Valley (GV) neighborhoods, as out of the seven wine samples, two-to-three were misclassified (Figure 2); e.g., two wines from the GV group and three wines from the MR group were classified as “none”, indicating that their elemental fingerprints did not match any of the established classes/neighborhood models. Interestingly, the three misclassified wines from the SH neighborhood were not all placed into the “none” category, as one wine was incorrectly identified as a wine from the SP and another one as from the MR neighborhood. The rate of false negatives ranged from 57–71% for these classes. The lack of sensitivity for these neighborhoods could be due to the small sample sizes per neighborhood. 

The cumulative explained variance for the first three components of the SIMCA model for each class (i.e., neighborhood) was very high, ranging from 99.97% for GV and SH to 99.99% for LR, MR, and SP. This indicates that each class is separated from the others (Table 3). The sensitivity of the multiclass SIMCA model, expressed as the sensitivity or true positive rate, was 100% for all neighborhoods, indicating that the SIMCA model recognizes class members very well. The specificity or true negative rate varied between 73.3% for Middle Reach (MR) and 96.8% for the Laguna Ridge (LR) neighborhood, with all neighborhoods except for MR showing a specificity of over 90% (Table 3). Last, the prediction accuracy was over 94% for all classes, ranging from 94.7% for neighborhoods MR and SP and 100% for wines from Green Valley (GV) and Sebastopol Hills (SH) (Table 3).

Despite these limitations, interestingly, none of the EH wine samples were placed into any of the existing classes (Figure 2). This means the residual variance in the EH samples exceeded the upper limit of every class in the model, and thus, the EH wines were not assigned to a category. The SIMCA results further implied that the wines produced in the EH neighborhood had a distinct enough and different enough element profile compared to wines produced in the four other neighborhoods in the Russian River Valley AVA. Future studies are needed to confirm that the placement of the EH wines in the “none” category is indeed indicative of a distinct EH elemental fingerprint.

Although the overall sample size is small (*n* = 53), the obtained results are of high importance to the wine industry and food authenticity testing at large. The strengths of this study are: (i) the collection of wines from different sub-regions within a single AVA, (ii) the analyses of wines of two different harvests, (iii) the use of commercial wines while maintaining some control over the winemaking process known to affect the elemental profile, and (iv) the application of a SIMCA classification algorithm that has not been previously applied to wines.

Past studies have used other modeling applications to predict wine in a certain growing region. For example, Angus et al. used a stepwise linear discriminant analysis to build a model to predict if one specific winegrowing district (Gimblett Gravels) could be distinguished from other wines produced in Hawke’s Bay [36]. SIMCA may be a better method for authenticity purposes, as the modeling procedure does not force samples into the modeled classes. That means that one does not need to know what the other classes may be, but rather, the classification algorithm is based on answering the question of whether the observed pattern is similar enough to be within the class boundaries or outside. For authenticity testing, answering this question is more appropriate, as one only needs to build a model of “within” samples. In our case, we were able to test the SIMCA algorithm for its appropriateness as we knew the EH samples came from a different sub-region in the Russian River Valley AVA, and we wanted to see if the model could be used to separate this new neighborhood from the old ones.

As indicated previously, the major limitation of this initial survey is the low number of wine samples from each neighborhood collected from the different wineries. Although wineries are known to change the elemental compositions (e.g., [26]), the effect of the individual winery was minimized by (i) collecting samples directly from fermentation vessels prior to any filtration and/or fining steps, (ii) comparing neighborhoods comprised of multiple wineries per neighborhood, and (iii) using data from two vintages. Although collected over two separate vintages, thus providing increased validity, future studies should collect more samples across multiple vintages and in metal-free sampling containers from each neighborhood, as well as from outside the established neighborhoods, to further refine the classification model. Eventually, vineyard attributes such as soil characteristics, rootstock, clone, and vine age might also provide deeper insights into the reasons for the differences within each neighborhood.

It is unknown whether and, if so, how much these existing elemental differences contribute to any perceivable flavor differences in the finished wines. Finally, a better understanding of the variability within the wine production process is needed to test whether the elemental fingerprints of finished wines, i.e., wines that have been fined, blended, bottled, and stored, would similarly be grouped by neighborhood. Similarly, elemental analyses of the vineyard soil and water used in the vineyard and winery, as well as any other viticultural and/or enological materials, could provide insights into the underlying causes for the observed, distinct elemental patterns by neighborhood.

## 3. Materials and Methods

Since this study was done over the course of 4 years, there were slight changes to the methods used to analyze the wine samples from the two vintages, e.g., different analysts and slight method modifications and improvements based on the advancements of the lab. Data validity was checked and ensured through the use of certified reference materials and spiking experiments for all employed analytical methods.

### 3.1. Materials and Reagents

A total of 53 commercial Pinot noir wines from single vineyard lots located within the Russian River Valley AVA in Bordeaux region, Northern CA, USA, were used in this study. Samples were aged between 4–5 months in neutral oak barrels and unfiltered, unfined, and not treated for physical instabilities. Samples were collected from each Russian River Valley neighborhood based on the local initiative aiming to understand the Pinot noir diversity in the AVA [43]; in each neighborhood, between 4 (EH) to 16 (GV) wineries produce Pinot noir wines. Samples were collected from the 2015 and 2017 vintages and were equally dispersed across the neighborhoods, with 5 wines each from the Green Valley (GV), Laguna Ridge (LR), Middle Reach (MR), Sebastopol Hills (SH), and Santa Rosa Plain (SP) neighborhoods (https://russianrivervalley.org/discover/neighborhoods). In 2017, another three wines were obtained from Eastern Hills (EH), which has been added as an additional neighborhood after the beginning of this study.

All sampled wines were transferred to the Food Safety & Measurement Facility at the University of California, Davis (Davis, CA, USA) where they were stored at 4 °C until analysis. In 2015, samples were transferred in sterile 50-mL plastic centrifuge tubes (VWR, Radnor, PA, USA) taken directly from fermentation vessels, while the 2017 harvest wines were stored in glass bottles.

Multielement standards (1, 2A, 3, and 4) and single element standards (P and Mn) were obtained from SPEX CertiPrep (Metuchen, NJ, USA). Single element standards for B and S were obtained from High Purity Standards (Charleston, SC. USA), while Si, Rb, and Sr were purchased from Crescent Chemical Company, Inc. (Islandia, NY, USA). Items purchased from Agilent Technologies (Santa Clara, CA, USA) included ionization buffer solution (100,000 mg/L Cs); two internal standard solutions (Internal Standard Mix (10-mg/L Bi, Ge, In, Li, Sc, Tb, and Y) and ICP internal standard (100 mg/L Li, Sc, Y, In, Tb, and Bi)); Environmental Spike Mix; Calibration Mix Majors; and single element standards Mg and K. All samples, quality controls, and calibration standards were prepared with ultrapure water (18 MΩ cm, EMD Millipore, Bellerica, MA, USA). Ethanol (200 proof) was obtained from Koptec (King of Prussia, PA, USA), and optima-grade nitric acid (HNO_3_, 67–69%) and hydrochloric acid (HCl, 32–35%) were purchased from Fisher Science (Pittsburg, PA, USA). Due to the lack of a wine-certified reference material (CRM), NIST 1643 (e/f) (Gaithersburg, MD, USA) and CRM-TMDW-B and CRM-TMDW (High Purity Standards) were used and adjusted to account for the ethanol sample matrix. Element-free centrifuge tubes (15 and 50 mL) were purchased from VWR (Radnor, PA, USA).

### 3.2. Macroelemental Profiling with Microwave Plasma-Atomic Emission Spectroscopy (MP-AES)

For the higher concentrated elements (B, Ca, Fe, K, Mg, Mn, Na, P, Rb, Si, and Sr), microwave plasma-atomic emission spectroscopy (4200 MP-AES; Agilent Technologies, Santa Clara, CA, USA) was used for the 2015 samples. Samples were diluted 5-fold with 5% HNO_3_ (*w*/*w*) and mixed with ICP internal standard solution and ionization buffer solution (diluted to 2000 mg/L with 1% HNO_3_ before use) in a mixing tee prior to entering a micromist nebulizer and a double-pass cyclic spray chamber. Matrix-matched calibration curves prepared in 4% ethanol and 5% HNO_3_ were established between 0.01 and 50 mg/kg (B, Ca, Fe, Na, Rb, Si, and Sr); 1 and 200 mg/kg (Mg); 0.01 and 2 mg/kg (Mn); and 1 and 700 mg/kg (K and P). Drinking water reference standard CRM-TMDW-B was diluted 3-fold in 4% ethanol and 5% HNO_3_, and sample spikes at two concentrations (low: 10 mg/kg and high: 100 mg/kg) were analyzed throughout the sample sequence to monitor the instrumental drift. Sample blanks of 4% ethanol and 5% HNO_3_ were used for the continuing calibration blank (CCB) and 1-mg/kg (10 mg/kg for Mg and 50 mg/kg for K/P) standards served as the continuing calibration verification (CCV) samples. Each wine was prepared in triplicate, and each sample was analyzed in triplicate, whereby each element was monitored with two or three different emission wavelengths. Wavelengths used for quantitation were free of interferences and chosen based on previous reports (Supplementary Table S1) [31]. Limits of detection (LOD) and quantitation (LOQ) were calculated according to Thomsen et al. [47].

### 3.3. Elemental Profiling with Inductively Coupled Plasma-Mass Spectrometry (ICP-MS)

Quantification of trace and microelements, covering the *m/z* range from 6 to 238, was completed using a triple-quadrupole ICP-MS (8800 ICP-MS; Agilent Technologies, Santa Clara, CA, USA). All wines were diluted 3-fold in 5% HNO_3_ (*v*/*v*) prior to analysis and mixed in a mixing tee with the Internal Standard Mix (diluted 10-fold in 1% HNO_3_ to 1 mg/kg) before entering the micromist nebulizer (Agilent Technologies; Santa Clara, CA, USA), with the quartz glass spray chamber held at 4 °C. Elements were monitored in no gas, helium (flow rate = 5mL/min (year 1) and 4.3 mL/min (year 2)), high-energy helium (flow rate = 10 mL/min), or oxygen mode (flow rate = 30%) (Supplementary Table S1). In the 8800 ICP-MS, the two quadrupoles are separated by an ORS^3^ collision/reaction cell. For certain elements, this tandem MS configuration allows for the control of interferences in the oxygen reaction mode, delivering greater accuracy. Each sample was diluted in triplicate, and each triplicate was measured in triplicate with 100 sweeps per replicate.

Matrix-matched (4% ethanol and 5% HNO_3_) 6-point calibration curves for each element were established between 0.1 and 500 µg/kg using multielement standards. Two different drinking water CRMs were diluted 10-fold in 4% ethanol and 5% HNO_3_ (CRM-TMDW and NIST 1643e) and analyzed throughout the sequence for quality control. In addition, samples were spiked with Environmental Spike Mix at two concentrations (low: 8.3 µg/kg and high: 83.3 µg/kg) and analyzed throughout the sample sequence. Sample blanks and 1-µg/kg calibration points served as CCBs and CCVs.

The monitored elemental isotopes were quantified using the MassHunter ICP-MS software (version 4.5, Agilent Technologies, Santa Clara, CA, USA). A specific gas mode and isotope were selected for each element based on the limits of detection (LOD) and reported the background equivalent concentrations (BEC) (Supplementary Table S1).

For the 2017 analyses, the method of analysis was slightly changed. Trace and microelement profiling were still completed with ICP-MS, as well as quantification of the major elements after a larger sample dilution. Furthermore, hydrochloric acid was introduced to all diluents to help stabilize some elements in the sample solution and to mitigate memory effects. For trace and microelement profiling, samples were first diluted 5-fold with 3% HNO_3_ and 1% HCl in metal-free centrifuge tubes. Furthermore, all calibration curves were prepared with at least 6 calibration points for each element, which began at 0.05 µg/kg. All other instrument parameters were the same as listed in the section above. In order to analyze the elements with high concentrations (> 2 mg/kg; Ca, Na, Mg, K, S, and P), wines were further diluted 250-fold and analyzed. Each wine was prepared and analyzed in triplicate at the two different dilution levels. The diluted samples were stored in 4 °C and centrifuged prior to analysis.

All calibration standards, blanks, and CRMs were made with a matrix-matched solution of 3% HNO_3_, 1% HCl, and 3% ethanol when analyzing the 5-fold diluted wine samples. Since the percentage of ethanol in the higher diluted samples was negligible, ethanol was not included in the calibration standards, blanks, and CRMs solutions while analyzing the higher diluted samples. In all cases, 6-point calibration curves were created for all quantified elements (Supplementary Table S1). Limits of detection (LOD) and quantitation (LOQ) were calculated according to Thomsen [47].

### 3.4. Data Treatment and Statistical Methods

For statistical analysis, elements that were not detected in any of the wine samples were removed from the analysis. Elemental concentrations below the LOD were set to LOD/100 for the multivariate data analyses. Multivariate analysis of variance (MANOVA) was conducted to test for significant differences among the neighborhoods (*p* < 0.05) in the two harvest years. Analysis of variance (ANOVA) for each element (*p* < 0.05) was then performed considering neighborhoods within the Russian River Valley AVA as factors. All analyses were done with R (Version 3.5.1, R Core Team) using RStudio (version 1.1.456, RStudio, Inc., Boston, MA, USA), with the additional packages *car*, [48] *emmeans*, [49] and *multcomp* [50] for the Tukey’s adjusted post-hoc comparisons of means. All elements that differed significantly between neighborhoods were further used in the canonical variate analysis (CVA, *candisc* package) [51].

Further testing was completed using the Soft Independent Modeling of Class Analogy (SIMCA), which was performed using the *mdatools* package [52]. SIMCA is used to identify possible groups within a dataset, create local models for each group, and then predict if new observations fit into the class models or not. A global principal component analysis is first completed to identify possible groups in the observations. Local models are then estimated for each class. If a new sample exceeds the residual variance for every model, it is not assigned to a class due to the assumption that the new sample is either an outlier or comes from a class not found in the dataset. SIMCA was used to determine whether the newly added neighborhood Eastern Hills (EH) would be separated from the other neighborhoods based on their unique elemental profiles. 

## Figures and Tables

**Table 1:** Mean elemental concentrations and lower and upper 95% confidence intervals for the 2015 vintage wines (*n* = 25) from the different neighborhoods (Green Valley: GV, Laguna Ridge: LR, Middle Reach: MR, Santa Rosa Plains: SP, and Sebastopol Hills: SH). Concentrations that share the same letters across rows are not significantly different according to Tukey’s post-hoc comparison (*p* < 0.05). Concentrations below the limits of detection (LOD) are reported as no detection (N.D.) and below the LOQ as < LOQ.

	GV (μg/kg)	LR (μg/kg)	MR (μg/kg)	SH (μg/kg)	SP (μg/kg)
Li	5.39 ab [2.91,8.58]	4.60 a [< 2.02,7.8]	2.83 a [N.D.,6.03]	3.65 a [N.D.,6.84]	3.65 a [6.47,12.9]
V	0.35 ab [0.18,0.51]	0.18 a [N.D.,0.34]	0.53 b [0.36,0.70]	0.29 ab [N.D.,0.46]	0.29 ab [0.36,0.70]
Co	6.51 b [4.53,8.49]	10.7 c [8.76,12.7]	2.33 a [0.35,4.31]	6.40 b [4.42,8.38]	6.40 b [2.12,6.08]
Ni	31.4 ab [21.0,41.7]	62.7 d [52.3,73.0]	24.4 a [14.0,34.8]	42.1 bc [31.7,52.5]	42.1 bc [44.9,65.7]
Ga	0.09 b [0.06,0.13]	0.06 ab [0.03,0.09]	0.09 ab [0.05,0.12]	0.04 a [< 0.02,0.08]	0.04 a [0.06,0.12]
Mo	1.75 b [0.83,2.66]	0.81 ab [N.D.,1.73]	0.36 a [N.D.,1.27]	0.56 ab [N.D.,1.48]	0.56 ab [N.D.,1.32]
Cd	0.22 d [0.19,0.26]	0.15 bc [0.12,0.19]	0.07 a [0.03,0.11]	0.16 c [0.12,0.20]	0.16 c [0.06,0.13]
Sb	0.09 b [0.06,0.13]	0.05 ab [< 0.03,0.08]	< 0.03 a [N.D.,0.05]	0.05 ab [< 0.03,0.08]	0.05 ab [N.D.,0.06]
Cs	5.58 a [1.88,9.29]	3.41 a [N.D.,7.11]	4.28 a [0.57,7.98]	2.43 a [N.D.,6.14]	2.43 a [11.1,18.5]
Ba	324 abc [231,418]	422 bc [328,515]	234 a [141,327]	298 ab [205,391]	298 ab [363,549]
Ce	0.08 b [0.05,0.10]	0.08 b [0.05,0.10]	0.03 a [N.D.,0.05]	0.03 a [0.01,0.06]	0.03 a [0.02,0.07]
Nd	0.07 c [0.05,.0.09]	0.06 bc [0.04,0.08]	0.02 a [N.D.,0.04]	0.03 a [N.D.,0.05]	0.03 a [0.01,0.06]
Ta	0.27 b [0.17,0.37]	0.01 a [N.D.,0.10]	N.D. a [N.D.,0.10]	0.05 ab [N.D.,0.15]	0.05 ab [N.D.,0.14]
W	0.31 b [0.18,0.43]	0.09 a [N.D.,0.22]	0.15 ab [0.02,0.28]	0.13 ab [N.D.,0.26]	0.13 ab [0.16,0.41]
Tl	0.52 b [0.32,0.72]	0.35 ab [0.15,0.55]	0.14 a [N.D.,0.34]	0.38 ab [0.18,0.58]	0.38 ab [0.20,0.60]
Pb ^1^	6.25 c [4.28,8.23]	3.06 ab [1.09,5.04]	1.40 a [N.D.,3.38]	5.22 bc [3.24,7.19]	5.22 bc [0.99,4.94]
	**GV (mg/kg)**	**LR (mg/kg)**	**MR (mg/kg)**	**SH (mg/kg)**	**SP (mg/kg)**
P	300 a [263,337]	298 a [261,335]	270 a [233,307]	304 ab [267,341]	356 b [319,393]
B	4.96 a [3.49,6.44]	5.80 ab [4.32,7.27]	8.44 c [6.97,9.91]	4.01 a [2.53,5.48]	7.70 bc [6.22,9.17]
Si	21.4 a [17.8,25.1]	18.1 a [14.5,21.7]	16.8 a [13.2,20.5]	19.2 a [15.6,22.8]	27.3 b [23.6,30.9]
Ca	60.3 b [54.8,65.8]	45.8 a [40.3,51.2]	50.20 a [44.7,55.6]	49.6 a [44.1,55.1]	49.1 a [43.6,54.5]
Mn	3.15 b [2.50,3.79]	2.65 b [2.00,3.29]	1.51 a [0.87,2.16]	2.71 b [2.06,3.36]	2.45 ab [1.80,3.10]
Sr	0.99 ab [0.73,1.26]	1.08 b [0.82,1.34]	0.83 ab [0.57,1.09]	1.58 c [1.32,1.84]	0.64 a [0.38,0.9]
K	467 a [410,525]	490 ab [432,548]	557 bc [499,614]	526 ab [468,583]	620 c [563,678]
Rb	1.42 ab [0.89,1.96]	1.62 b [1.08,2.15]	0.75 a [0.22,1.29]	1.36 ab [0.83,1.89]	2.48 c [1.95,3.02]

^1^ Mean of concentrations of the Pb 206, 207, and 208 isotopes.

**Table 2:** Mean elemental concentrations and lower and upper 95% confidence intervals for the 2017 vintage wines (*n* = 27) from the different neighborhoods (Eastern Hills: EH, Green Valley: GV, Laguna Ridge: LR, Middle Reach: MR, Santa Rosa Plains: SP, and Sebastopol Hills: SH). Concentrations that share the same letters across rows are not significantly different according to Tukey’s post-hoc comparison (*p* < 0.05). Concentrations below the LOD are reported as N.D. and below the LOQ as < LOQ.

	GV (μg/kg)	LR (μg/kg)	MR (μg/kg)	SH (μg/kg)	SP (μg/kg)	EH (μg/kg)
Li	6.02 ab [< 0.47,11.9]	5.49 ab [N.D.,11.4]	12.1 b [6.22,18.0]	1.95 a [N.D.,7.82]	8.03 ab [2.16,13.9]	6.35 ab [N.D.,13.9]
B	6286 ab [4994,7578]	6604 ab [5312,7896]	7507 b [6215,8800]	5255 a [3963,6548]	7651 b [6359,8943]	5538 ab [3870,7207]
Al	204 bc [168,240]	131 a [94.6,167]	226 bc [190,262]	222 bc [186,258]	201 b [164,237]	264 c [218,311]
Ti	17.4 b [16.0,18.8]	12.6 a [11.2,14.0]	12.7 a [11.3,14.1]	13.2 a [11.8,14.6]	12.0 a [10.6,13.5]	14.4 a [12.6,16.2]
V	< 0.01 a [N.D.,0.14]	< 0.01 a [N.D.,0.14]	< 0.01 a [N.D.,0.14]	0.22 b [0.09,0.36]	< 0.01 a [N.D.,0.14]	< 0.01 ab [N.D.,0.18]
Cr	4.24 bc [2.70,5.78]	1.55 a [< 0.20,3.09]	5.96 c [4.42,7.50]	3.24 ab [1.70,4.78]	3.80 abc [2.26,5.34]	5.05 bc [3.06,7.04]
Mn	1892 ab [1343,2442]	1740 ab [1190,2289]	1474 a [925,2024]	2109 ab [1559,2658]	2002 ab [1453,2552]	2451 b [1741,3160]
Fe	684 a [411,956]	723 a [450,995]	1636 b [1324,1909]	1244 bc [972,1517]	1151 b [879,1424]	9412 ab [590,1294]
Co	4.19 ab [2.37,6.00]	5.50 b [3.69,7.31]	2.20 a [0.38,4.01]	4.52 ab [2.70,6.33]	2.79 ab [0.98,4.60]	5.36 ab [3.02,7.70]
Ni	24.8 a [16.9,32.8]	34.7 ab [26.8,42.6]	32.1 a [24.2,40.0]	30.8 a [22.9,38.7]	45.4 bc [37.4,53.3]	54.3 c [44.1,64.5]
Cu	29.4 a [1.36,57.4]	44.5 a [16.5,72.5]	45.3 a [17.2,73.3]	52.5 ab [24.5,80.5]	92.8 b [64.8,121]	29.0 a [N.D.,65.2]
As	1.36 b [0.53,2.20]	2.25 b [1.41,3.08]	< 0.17 b [N.D.,0.84]	1.13 ab [0.30,1.96]	1.07 ab [0.24,1.91]	0.91 ab [N.D.,1.99]
Rb	1842 ab [1542,2232]	1468 a [1078,1857]	1404 a [1015,1794]	1809 ab [1420,2199]	2346 b [1956,2736]	2075 ab [1572,2578]
Sr	852 a [609,1094]	750 a [507,992]	702 a [460,945]	1558 b [1316,1801]	573 a [331,816]	675 a [362,988]
Zr	2.96 b [2.44,3.49]	1.43 a [0.91,1.96]	1.87 a [1.34,2.40]	2.91 b [2.39,3.44]	1.43 a [0.91,1.96]	2.88 b [2.20,3.56]
Mo	4.75 ab [0.66,8.85]	0.22 a [N.D.,4.31]	6.56 b [2.47,10.7]	0.29 a [N.D.,4.38]	2.68 ab [N.D.,6.77]	N.D. ab [N.D.,5.28]
La	0.06 b [0.03,0.09]	0.05 ab [0.02,0.07]	0.02 a [N.D.,0.04]	0.03 ab [0.01,0.06]	0.06 ab [0.03,0.08]	0.07 b [0.03,0.10]
Ce	0.11 b [0.06,0.15]	0.08 ab [0.03,0.12]	0.03 a [N.D.,0.08]	0.11 b [0.06,0.16]	0.09 ab [0.04,0.13]	0.12 b [0.06,0.18]
Nd	0.05 b [0.03,0.08]	0.05 b [0.03,0.07]	0.01 a [N.D.,0.03]	0.02 ab [N.D.,0.04]	0.04 ab [0.02,0.06]	0.05 ab [0.02,0.07]
Gd	0.02 abc [N.D.,0.03]	0.02 bc [N.D.,0.03]	<0.02 a [N.D.,<0.02]	<0.02 ab [N.D.,0.02]	0.02 abc [N.D.,0.02]	0.03 c [N.D.,0.04]
Dy	0.02 a [0.01,0.04]	0.03 ab [0.01,0.04]	0.01 a [N.D.,0.03]	0.01 ab [N.D.,0.03]	0.03 a [0.01,0.04]	0.05 b [0.03,0.07]
Er	0.03 bc [0.02,0.04]	0.03 abc [0.01,0.04]	0.01 ab [N.D., 0.02]	0.01 ab [N.D.,0.02]	0.03 abc [0.01,0.04]	0.05 c [0.03,0.06]
Yb	0.04 b [0.03,0.05]	0.03 ab [0.02,0.04]	<0.01 a [<0.01,0.03]	0.02 a [<0.01,0.03]	0.03 ab [0.02,0.04]	0.04 b [0.03,0.06]
Hf	0.13 b 0.09,0.17]	0.07 a [0.03,0.11]	0.10 ab [0.06,0.14]	0.13 ab [0.09,0.17]	0.08 ab [0.04,0.12]	0.14 ab [0.09,0.19]
W	0.03 ab [0.03,0.04]	0.03 a [0.02,0.04]	0.05 b [0.04,0.05]	0.03 ab [0.02,0.04]	0.04 ab [0.03,0.05]	0.04 ab [0.03,0.05]
Pb ^1^	3.40 c [2.15,4.65]	1.44 ab [0.19,2.69]	0.46 a [N.D.,1.71]	3.08 bc [1.83,4.33]	1.86 abc [0.61,3.11]	2.99 bc [1.38,4.60]
	**GV (mg/kg)**	**LR (mg/kg)**	**MR (mg/kg)**	**SH (mg/kg)**	**SP (mg/kg)**	**EH (mg/kg)**
Mg	123 a [108,138]	132 ab [117,147]	127 a [112,142]	133 ab [118,148]	124 a [109,139]	156 b [137,175]
K	913 ab [788,1037]	790 a [665,914]	993 b [868,1117]	855 ab [730,979]	1022 b [898,1147]	984 ab [823,1145]
Ca	73.8 b [66.6,81.0]	53.5 a [48.2,62.6]	55.4 a [48.2,62.6]	58.9 a [51.7,66.1]	50.0 a [42.8,57.2]	59.6 a [50.3,68.9]
Si	28.5 a [25.8,31.1]	18.6 a [16.0,21.3]	21.7 a [19.0,24.3]	25.8 b [23.1,28.4]	26.3 b [23.6,28.9]	34.1 c [30.7,37.5]
S	118 ab [98.8,136]	112 ab [93.5,131]	130 b [111,149]	107 ab [88.0,126]	99.1 a [80.3,118]	85.0 a [60.7,109]

^1^ Mean of the concentrations of the Pb 206, 207, and 208 isotopes.

**Table 3:** Multiclass Soft Independent Modeling of Class Analogies (SIMCA) model for the neighborhoods Green Valley (GV), Laguna Ridge (LR), Middle Reach (MR), Sebastopol Hills (SH), and Santa Rosa Plain (SP). Critical limits and outlier limits were set to 0.05 and 0.01, respectively.

	Classes				
	GV	LR	MR	SH	SP
Model components	3	3	3	3	3
Cumulative variance (%)	99.97	99.99	99.99	99.97	99.99
Specificity (%)	96.7	96.8	73.3	90.3	96.7
Sensitivity (%)	100	100	100	100	100
Accuracy (%)	100	97.4	94.7	100	94.7

## Supplementary Materials

The following are available online: Table S1. Calibration curve parameters for the elemental measurements.
