# Supplementary material for: The Use of Macro, Micro, and Trace Elemental Profiles to Differentiate Commercial Single Vineyard Pinot noir Wines at a Sub-Regional Level
Source: Molecules. 2020 May 30;25(11):2552. doi: 10.3390/molecules25112552 (PMC7321060; doi:10.3390/molecules25112552)
Supplement: Supplementary file 1 [file molecules-25-02552-s001.pdf]

Table S1. Calibration curve parameters for all elements as analyzed by **(a)** ICP-MS and MP-AES for vintage 2015, and **(b)** ICP-MS for vintage 2017.

(a)	Element	LOD (µg/kg)	LOQ (µg/kg)	R^2	Element	LOD (µg/kg)	LOQ (µg/kg)	R^2	Element	LOD (µg/kg)	LOQ (µg/kg)	R^2
ICP-MS	7Li	0.6068	2.0228	0.9998	103Rh	0.0005	0.0016	1.0000	166Er	0.0005	0.0018	1.0000
	27Al	0.8100	2.7000	1.0000	105Pd	0.0212	0.0707	0.9988	169Tm	0.0003	0.0008	1.0000
	47Ti	0.3118	1.0393	0.9991	107Ag	0.0105	0.0351	0.9997	172Yb	0.0026	0.0086	1.0000
	51V	0.0126	0.0419	1.0000	111Cd	0.0068	0.0228	1.0000	175Lu	0.0079	0.0264	1.0000
	52Cr	0.0828	0.2759	1.0000	123Sb	0.0099	0.0329	1.0000	178Hf	0.0189	0.0631	0.9999
	59Co	0.0066	0.0221	1.0000	125Te	0.0000	0.0000	0.9999	181Ta	0.0020	0.0068	1.0000
	60Ni	0.0541	0.1804	1.0000	133Cs	0.0050	0.0165	1.0000	182W	0.0048	0.0159	0.9999
	65Cu	0.0401	0.1336	1.0000	137Ba	0.0435	0.1449	1.0000	185Re	0.0019	0.0065	0.9999
	66Zn	0.0904	0.3014	1.0000	141Pr	0.0007	0.0023	1.0000	193Ir	0.0025	0.0083	1.0000
	71Ga	0.0059	0.0195	1.0000	142Ce	0.0015	0.0049	1.0000	195Pt	0.0011	0.0036	1.0000
	75As	0.0081	0.0269	1.0000	146Nd	0.0027	0.0088	1.0000	197Au	0.0268	0.0895	0.9970
	78Se	0.0313	0.1044	1.0000	147Sm	0.0007	0.0023	1.0000	205Tl	0.0014	0.0045	1.0000
	90Zr	0.0281	0.0936	0.9997	153Eu	0.0008	0.0025	1.0000	*Pb	0.0045	0.0150	1.0000
	93Nb	0.0030	0.0100	1.0000	157Gd	0.0007	0.0023	1.0000	232Th	0.0014	0.0048	1.0000
	98Mo	0.0270	0.0900	1.0000	163Dy	0.0002	0.0007	1.0000	238U	0.0010	0.0034	1.0000
	101Ru	0.0066	0.0221	1.0000	165Ho	0.0002	0.0008	1.0000				
	Element	LOD (mg/kg)	LOQ (mg/kg)	R^2	Element	LOD (mg/kg)	LOQ (mg/kg)	R^2	Element	LOD (mg/kg)	LOQ (mg/kg)	R^2
MP-AES	214.915 P	2.1939	43.8784	0.9994	371.993 Fe	0.0074	0.1479	1.0000	589.592 Na	0.0246	0.4911	0.9993
	249.677 B	0.0027	0.0547	0.9998	396.847 Ca	0.0030	0.0608	0.9998	769.897 K	0.0108	0.2160	0.9991
	251.611 Si	0.0171	0.3429	0.9999	403.076 Mn	0.0081	0.1620	0.9997	780.027 Rb	0.0324	0.6480	0.9998
	285.213 Mg	0.0015	0.0293	0.9963	421.552 Sr	0.0013	0.0254	1.0000				
(b)	Element	LOD (µg/kg)	LOQ (µg/kg)	R^2	Element	LOD (µg/kg)	LOQ (µg/kg)	R^2	Element	LOD (µg/kg)	LOQ (µg/kg)	R^2
ICP-MS (1:5)	7Li	1.5764	5.2546	0.9997	90Zr	0.0087	0.0290	0.9998	157Gd	0.0058	0.0192	1.0000
	9Be	3.3223	11.0745	1.0000	93Nb	0.0016	0.0054	0.9992	163Dy	0.0040	0.0134	1.0000
	11B	0.4800	1.6001	0.9999	98Mo	0.0129	0.0429	1.0000	165Ho	0.0020	0.0066	1.0000
	27Al	2.8000	9.3335	1.0000	101Ru	0.0037	0.0124	1.0000	166Er	0.0030	0.0098	1.0000
	48Ti	0.0188	0.0628	0.9999	103Rh	0.0028	0.0094	1.0000	169Tm	0.0013	0.0044	1.0000
	51V	0.7866	2.6221	0.9998	105Pd	0.0100	0.0335	0.9979	172Yb	0.0043	0.0143	1.0000
	52Cr	2.4604	8.2012	0.9999	107Ag	0.4599	1.5331	1.0000	175Lu	0.0126	0.0419	1.0000
	55Mn	2.2480	7.4932	1.0000	111Cd	1.0941	3.6469	1.0000	178Hf	0.0072	0.0241	1.0000
	56Fe	2.2534	7.5113	1.0000	121Sb	0.0062	0.0206	1.0000	181Ta	0.0021	0.0072	1.0000
	59Co	2.1096	7.0319	1.0000	125Te	0.0206	0.0686	1.0000	182W	0.0038	0.0125	1.0000
	60Ni	2.0193	6.7311	1.0000	133Cs	1.1601	3.8672	1.0000	185Re	0.0038	0.0126	1.0000
	63Cu	2.1613	7.2045	1.0000	138Ba	1.4816	4.9385	1.0000	193Ir	0.0031	0.0103	1.0000
	66Zn	1.1509	3.8363	0.9999	139La	0.0019	0.0062	1.0000	195Pt	0.0034	0.0112	1.0000
	71Ga	1.2738	4.2459	1.0000	140Ce	0.0016	0.0052	1.0000	197Au	0.0125	0.0417	0.9997
	75As	0.9334	3.1113	1.0000	141Pr	0.0021	0.0069	1.0000	205Tl	1.3815	4.6050	0.9992
	80Se	0.7555	2.5185	1.0000	146Nd	0.0043	0.0143	1.0000	*Pb	1.5949	5.3163	1.0000
	85Rb	1.3523	4.5076	1.0000	147Sm	0.0027	0.0090	1.0000	232Th	0.0016	0.0054	1.0000
	88Sr	1.6129	5.3762	1.0000	153Eu	0.0023	0.0076	1.0000	238U	1.1651	3.8838	0.9993
	Element	LOD (µg/kg)	LOQ (µg/kg)	R^2	Element	LOD (µg/kg)	LOQ (µg/kg)	R^2	Element	LOD (µg/kg)	LOQ (µg/kg)	R^2
ICP-MS (1:250)	23Na	0.4725	1.5749	1.0000	31P	1.1587	3.8623	1.0000	44Ca	2.3773	7.9245	0.9999
	26Mg	0.4333	1.4444	1.0000	32S	0.5906	1.9686	1.0000				
	28Si	5.9381	19.7938	0.9998	39K	1.9477	6.4924	1.0000				

*Average of concentrations found at for Pb 206, 207, and 208 isotopes
